# CDC167 exhibits potential as a biomarker for airway inflammation in asthma

**DOI:** 10.1007/s00335-024-10037-4

**Published:** 2024-04-05

**Authors:** Yukai Zhong, Qiong Wu, Li Cai, Yuanjing Chen, Qi Shen

**Affiliations:** 1 Department of Pediatrics, Kongjiang Hospital of Shanghai Yangpu District, Shanghai, 200093 China; 2 Department of Respiratory, Kongjiang Hospital of Shanghai Yangpu District, No. 480 Shuang Yang Road, Yangpu District, Shanghai, 200093 China; 3 Department of Colorectal Surgery, Kongjiang Hospital of Shanghai Yangpu District, Shanghai, 200093 China; 4 Department of Geriatric Medicine, Tongji University Affiliated Yangpu Hospital, No. 450 Teng Yue Road, Yangpu District, Shanghai, 200090 China

## Abstract

Current asthma treatments have been discovered to decrease the risk of disease progression. Herein, we aimed to characterize novel potential therapeutic targets for asthma. Differentially expressed genes (DEGs) for GSE64913 and GSE137268 datasets were characterized. Weighted correlation network analysis (WGCNA) was used to identify trait-related module genes within the GSE67472 dataset. The intersection of the module genes of interest, as well as the DEGs, comprised the key module genes that underwent additional candidate gene screening using machine learning. In addition, a bioinformatics-based approach was used to analyze the relative expression levels, diagnostic values, and reverently enriched pathways of the screened candidate genes. Furthermore, the candidate genes were silenced in asthmatic mice, and the inflammation and lung injury in the mice were validated. A total of 1710 DEGs were characterized in GSE64913 and GSE137268 for asthma patients. WGCNA identified 2367 asthma module genes, of which 285 overlapped with 1710 DEGs. Four candidate genes, CDC167, POSTN, SEC14L1, and SERPINB2, were validated using the intersection genes of three machine learning algorithms, including Least Absolute Shrinkage and Selection Operator, Random Forest, and Support Vector Machine. All the candidate genes were significantly upregulated in asthma patients and demonstrated diagnostic utility for asthma. Furthermore, silencing CDC167 reduced the levels of inflammatory cytokines significantly and alleviated lung injury in ovalbumin (OVA)-induced asthmatic mice. Our study demonstrated that CDC167 exhibits potential as diagnostic markers and therapeutic targets for asthma patients.

## Introduction

Asthma is a highly prevalent chronic respiratory condition that is observed on a global scale. According to available data, there has been a decline in the estimated prevalence of asthma, with rates decreasing from 601.2 to 477.9 per 1,000,000 individuals between the years 1990 and 2019 (Cao et al. 2022). Nevertheless, the frequency of these incidents continues to increase, highlighting the ongoing need to address the burden of disease, particularly among school-aged children (Asher et al. 2021). The primary approach to managing asthma includes the use of short-acting β(2)-agonist (SABA) medications and inhaled corticosteroids (Fuhlbrigge and Sharma 2021; Nwaru et al. 2020). These treatments are intended to prevent or minimize asthma symptoms. However, it is important to note that the use of these medications has been associated with certain adverse effects, such as an increased likelihood of exacerbations and even higher mortality rates (Fuhlbrigge and Sharma 2021; Nwaru et al. 2020). The pathophysiology of asthma encompasses inflammation, hyperresponsiveness, and airway remodeling. However, the considerable heterogeneity of the disease makes it difficult to identify biomarkers and develop novel therapeutic approaches (Busse et al. 2022). In addition, the characterized subtypes are not consistently stable; for instance, Type-2 (T2 or Th2)-low asthma can transition to the T2-high subtype as the disease progresses, and vice versa, based on the T2 inflammation-based classification standards (Habib et al. 2022). As a result, there is an urgent need to characterize novel biomarkers and therapeutic targets in order to enhance the diagnosis and treatment of asthma.

The utilization of bioinformatics analysis has demonstrated significant promise in the characterization of biomarkers and therapeutic targets in various diseases, including asthma (Abdel-Aziz et al. 2020). Accordingly, ~ 38% of childhood asthma cases have been attributed to various hereditary factors, which have been associated with immune responses, muscle function, and lung function (Zayed 2020). Furthermore, the presence of disease heterogeneity, variations in datasets or sample sources, individual differences among patients, and differences in analytical methodology contribute to the variability observed in various studies. For example, weighted gene co-expression network analysis (WGCNA) was applied using the GSE43696 dataset, resulting in the identification and characterization of 15 hub genes. These hub genes include BIRC5, CCNB2, CDCA2, MELK, UBE2C, and KIF20A, among others (He et al. 2020). The GSE89809 dataset was subjected to comparable analyses, yielding hub genes such as CCR1, CCR7, CXCR1, CXCR2, TLR2, FCGR3B, and FPR1 (Zhang et al. 2021). However, these findings diverged significantly from previous analyses despite some shared characteristic biological processes. Additionally, the presence of heterogeneity was observed in various studies, necessitating the utilization of multiple datasets and comprehensive analytical methodologies to identify universal biomarkers and therapeutic targets for further characterization.

The objective of this study was to discover new potential biomarkers using three machine learning algorithms: Least Absolute Shrinkage and Selection Operator (LASSO), Random Forest, and Support Vector Machine (SVM). The differentially expressed genes (DEGs) from the GSE64913 and GSE137268 datasets were combined. These datasets consisted of samples collected from the airway epithelium and induced sputum of individuals diagnosed with asthma, respectively. The genes belonging to the module that was highly correlated with asthma were characterized using the GSE67472 dataset. In addition, the intersect genes of the module genes and the DEGs were analyzed with machine learning in order to identify hub genes associated with asthma. Furthermore, the hub genes were confirmed by examining their expression profiles in both the dataset and asthmatic mice. The findings demonstrate that CDC167, POSTN, SEC14L1, and SERPINB2 exhibit potential as diagnostic markers and therapeutic targets for individuals with asthma.

## Materials and methods

### Acquisition of asthma-related datasets

**Fig. 1:**
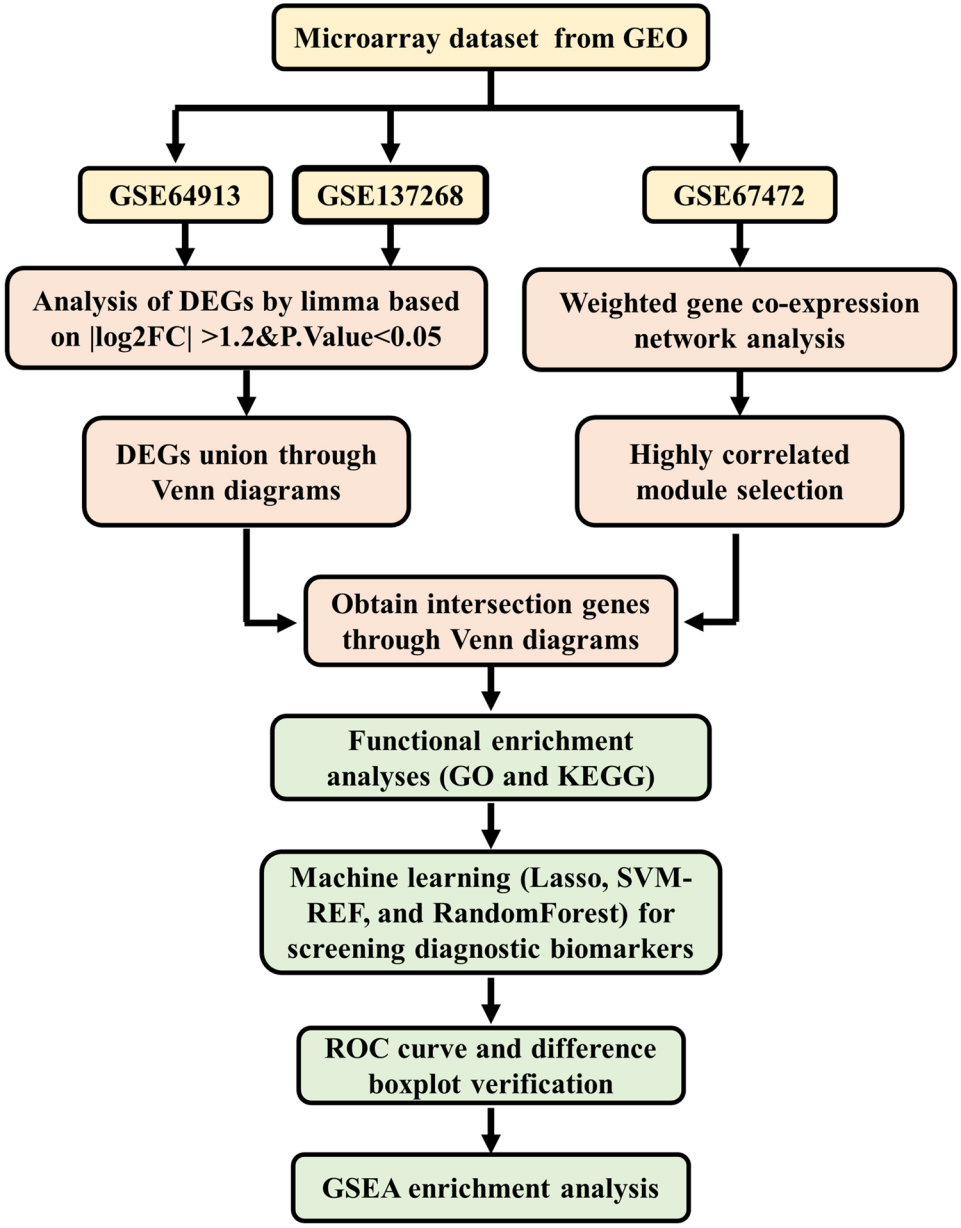
Workflow for the characterization of hub genes

The workflow of our bioinformatics analyses is summarized in Fig. 1. The datasets pertaining to asthma were obtained by downloading them from the GEO database, which identified differential gene expression and pathways in asthma and help us to find the potential as diagnostic markers, accessible at https://www.ncbi.nlm.nih.gov/geo/. The GSE64913 dataset comprises airway epithelial biopsy samples obtained from a cohort of 42 healthy individuals and 28 patients diagnosed with asthma. Similarly, the GSE67472 dataset encompasses epithelium samples collected from 43 healthy individuals and 62 asthma patients. Lastly, the GSE137268 dataset consists of induced sputum samples obtained from 15 healthy individuals and 54 patients diagnosed with asthma. The basic information of the GEO datasets was shown in Table 1.

**Table 1:** Basic information of the GEO datasets

GEO	Organism	Phenotype	Submission date	Platforms	Samples	Control	Asthma	Experiment type
GSE64913	Homo sapiens	Severe asthma	Mar 25, 2019	GPL570	70	42	28	Array
GSE67472	Homo sapiens	Mild-to-moderate asthma	May 06, 2021	GPL16311	105	43	62	Array
GSE137268	Homo sapiens	Sputum asthma	Sep 13, 2019	GPL6104	69	15	54	Array

### Characterization of DEGs

The “limma” package in the R software was used to screen the DEGs that were upregulated and downregulated in the GSE64913 and GSE137268 datasets. The screening criteria were set as |log2FC|> 1.2 and P value < 0.05. The volcano plot was generated using the “ggplot2” package. All the GEGs from both datasets were collected for subsequent analysis.

### Weighted correlation network analysis (WGCNA) analysis

According to the WGCNA method (Botía et al. 2017; Langfelder and Horvath 2008), the GSE67472 dataset with most varied of genes was subsequently employed to examine the genes that are most likely associated with asthma using. The “goodSamplesGenes” function from the “WGCNA” R package was employed to assess the presence of missing and discrete values. Subsequently, the “hclust” function was utilized in hierarchical cluster analysis to eliminate outliers and generate a heat map illustrating the correlations between modules and traits. To circumvent the issue of arbitrary thresholds, a soft threshold was employed, while ensuring scale independence and mean connectivity by keeping the powers fixed. When the soft threshold power was defined as 10, the scale-free topology index was 0.9. Thus, this network conformed to the power-law distribution and closer to the real biological network state (Yang et al. 2018). The process of gene clustering involved the utilization of the “TOMsimilarity” and “hclust” functions to create the weighted adjacency matrix and transformed topological overlap matrix (TOM). The “cutreeDynamic” function was used to self-adaptively prune the dynamically identified modules of the hierarchical clustering tree by setting 60 genes for each module. The co-expressed modules were generated using the “dynamicTreeCut” algorithm and subsequently clustered using the “moduleEigengenes” approach. The modules that exhibited similarities were subsequently subjected to clustering using the “hclust” function, with a height threshold of 0.25. Subsequently, the clinical correlations of the genes within the module were assessed using the “corPvalueStudent” function. Subsequently, gene significance and module membership were computed for each module. Subsequently, the module genes of interest, identified in both the previously characterized DEGs and the most prominent module associated with asthma, were selected for subsequent analysis.

### Gene ontology (GO) and kyoto encyclopedia of genes and genomes (KEGG) enrichment

The “clusterProfile” package was used to apply GO and KEGG enrichment to the DEGs as well as the relevant module genes. Included in GO enrichment are biological process (BP), cellular component (CC), and molecular function (MF). P value < 0.05 were applied to both enrichments.

### Machine learning

The LASSO, Random Forest, and SVM algorithms were utilized to identify hub genes among the module genes of interest. The module genes of interest were normalized with the DEGs. LASSO was a conventional feature selection method, which could screen important differential genes (Alhamzawi and Ali 2018). Random Forest could be highly parallelized to obtain a more accurate and stable model (Asadi et al. 2021). And SVM algorithms help with gene classification and regression tasks (Uddin et al. 2019). In addition, the R software’s “glmnet” function was used to conduct the analysis for LASSO linear regression. Subsequently, the “cv.glmnet” function was used for cross-model validation, and the “coef” function was used to analyze the optimized genes. For Random Forest analysis, R package “randomForest” was used to construct the forest, and the 30 most important asthma-related genes were selected as potential hub genes. For SVM, SVM-REF was constructed using the “caret” and “randomForest” R packages. Finally, the potential hub genes characterized by all three algorithms were deemed hub genes, which were characterized by the “VennDiagram” R package.

### ROC evaluation

The module genes of interest were normalized using the DEGs. Subsequently, the predictive value of the predicted hub genes was evaluated using ROC curves using the “pROC” R package. Subsequently, the area under the curve (AUC) and 95% confidence interval (CI) were calculated.

### Gene-set enrichment analysis (GSEA)

The R packages “clusterProfiler” and “org.Hs.eg.db” were utilized to perform GSEA in order to identify the signaling pathways associated with hub genes in all three characterized datasets.

### Animal experiments

In order to validate the hub gene CCDC167, an in vivo verification was conducted by referencing a previously published report (Wu et al. 2022). In this study, female BALB/c mice (aged 5 ± 1 weeks) were procured from the Experimental Animal Centre of East China Normal University [SCXK (Shanghai) 2021-0006] and subsequently housed in animal facilities that maintained specific pathogen-free conditions. All experimental procedures were reviewed and approved by the Animal Care and Use Committee of Kongjiang Hospital. After a period of acclimation lasting one week, a total of 10 mice were randomly assigned to one of three groups: the control group, which received treatment with saline; the shNC group, which induced asthma and received treatment with short hairpin (sh) RNA as a negative control; and the shCCDC167 group, which induced asthma and received treatment with shCCDC167. To induce asthma, a solution consisting of 20 μg ovalbumin (OVA, Sigma-Aldrich, St. Louis, MO) and 2 mg aluminum hydroxide (769,460, Sigma-Aldrich) was administered via intraperitoneal injection in a volume of 1 mL on day 7, day 14, and day 21. Subsequently, the mice with asthma were confined within a transparent enclosed cage measuring 20 × 20 × 30 cm for a duration spanning from day 27 to day 30. During this period, they were subjected to daily treatment with a 1% OVA solution administered via atomization for a duration of 20 min. The mice in the control group were administered a comparable quantity of normal saline. Furthermore, in the shNC and shCCDC167 experimental groups, a total volume of 50 μL of AAV6 recombinant vector (obtained from Genepharma Co., Ltd., Shanghai, China) containing either shNC or shCCDC167 was administered to the lungs of the mice via endotracheal intubation on day 1 and day 7. On the 34th day of the experiment, all mice were administered an intraperitoneal injection of pentobarbital sodium (100 mg/kg, Vetoquinol, Cedex, France). Subsequently, the bronchoalveolar lavage fluid (BALF) and lung tissues were collected for subsequent analysis. The shRNAs (Genepharma) employed in this study were as follows: CCDC167 shRNA: 5ʹ -CCG GCC TAG TGT TCA AGC ATG GCT TCT CGA GAA GCC ATG CTT GAA CAC TAG GTT TTT TG-3ʹ, and control shRNA: 5ʹ -CCG GCC TAG TGT TCA AGC ATG GCT TCT CGA GAA GCC ATG CTT GAA CAC TAG GTT TTT TG-3ʹ. The cytokines IgE (J24307, Giled Biotechnology, Wuhan, China), IL-4 (J24113, Giled), IL-5 (J24112, Giled), and IL-13 (J24122, Giled) present in bronchoalveolar lavage fluid (BALF) were examined using ELISA kits as per the manufacturer's guidelines. The mouse lung tissues were fixed using a 10% formalin solution, which was followed by dehydration, paraffin embedding, and subsequent slicing into sections measuring 5 μm in thickness. The sliced sections underwent regular dewaxing and staining procedures using hematoxylin and eosin (H&E), periodic acid-Schiff stain (PAS), and Masson's trichrome stain to observe the presence of inflammatory cell infiltration, smooth muscle cell hyperplasia, and airway mucus secretion in the samples. The measurements of the inner area of the bronchial wall (WAi), airway smooth muscle area (WAm), number of bronchial smooth muscle cells (N), and inner perimeter of the bronchial wall (Pi) were conducted using Image Pro Plus software (Media Cybernetics Inc., MD, USA). These measurements were obtained after the sections were observed at 40X magnification and photographed using a microscope (Olympus CX41, Tokyo, Japan).

### Statistics

Statistical analyses were conducted using R software (v4.2.1) and GraphPad Prism (V9.4.0, GraphPad Software, San Diego, CA, USA). Unless otherwise specified, the Student's t-test was employed for comparing between two groups, while one-way analysis of variance (ANOVA) was utilized for comparing among three or more groups. A P value < 0.05 was deemed to be statistically significant.

## Results

### Characterization of module genes

**Fig. 2:**
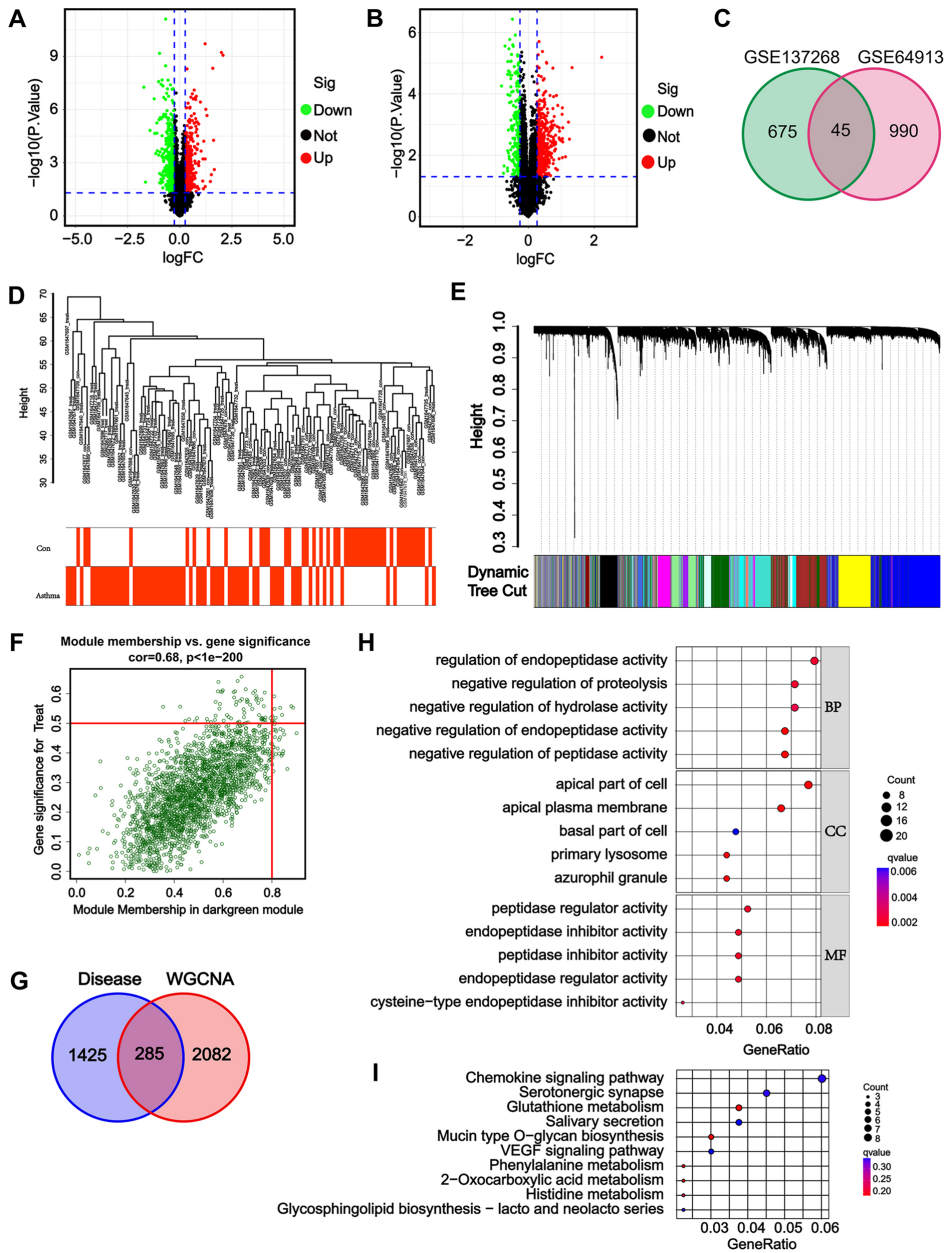
Characterization of module genes using WGCNA. **A** DEGs for GSE64913; **B** DEGs for GSE137268; **C** Overlap of DEGs in the GSE64913 and GSE137268 datasets; **D** Genes from GSE67472 dataset with similar expression patterns were clustered into different modules to detect outliers; **E** Sample clustering tree diagram; **F** Heat map of module-trait correlations; **G** Scatter plots showing correlation and importance between genes and modules for module dark green; **H** Overlapping of the dark green module with DEGs form GSE64913 and GSE137268 datasets; **I** Gene Ontology (GO) enrichment of the overlapped module genes; **J** Kyoto Encyclopedia of Genes and Genomes (KEGG) enrichment of the overlapped module genes

The DEGs for the GSE64913 and GSE137268 datasets, which include samples of airway epithelium and induced sputum, were characterized using the “limma” package of the R software. A total of 720 DEGs were identified for GSE137268, of which 488 were upregulated and 232 were downregulated (Fig. 2A). For GSE64913, 1035 DEGs were identified, of which 516 were upregulated and 519 were downregulated (Fig. 2B). DEGs for the two datasets were significantly different, with 1035 DEGs for GSE64913 and 720 DEGs for GSE137268; however, only 45 intersect genes were characterized between the two datasets (Fig. 2C). Then, subsequent analysis was conducted on all DEGs from both datasets. The GSE67472 dataset, which contains airway epithelium samples from healthy donors and asthma patients, was normalized using DEGs and analyzed using the WGCNA package. Resulting gene dendrograms and respective module colors were displayed in Fig. 2D and E. The disease-related modules with the greatest significance were identified (Fig. 2F). Among the characterized modules, the light-cyan, cyan, and salmon modules were significantly downregulated in asthma patients, whereas the purple, brown, and dark-green modules were significantly upregulated. The correlation and importance between genes and modules for the dark-green module are depicted in Fig. 2G; accordingly, 285 genes characterized both in the dark-green module and in the DEGs were chosen as the module genes of interest that were used in subsequent analyses (Fig. 2H). The module genes of interest were primarily enriched in negative regulation of proteolysis, hydrolase, peptidase and endopeptidase activities (BP), regulation of endopeptidase activity (BP), apical part of cell (CC), apical plasma membrane (CC), and peptidase activities (MF) by GO analysis (Fig. 2I). Furthermore, via KEGG enrichment, the genes of interest were predominantly enriched in the chemokine signaling pathway, serotonergic synapse, glutathione metabolism, and salivary secretion (Fig. 2J). Collectively, we characterized 285 module genes of interest for future analyses.

### Identification of asthma-related hub genes via machine learning algorithms

**Fig. 3:**
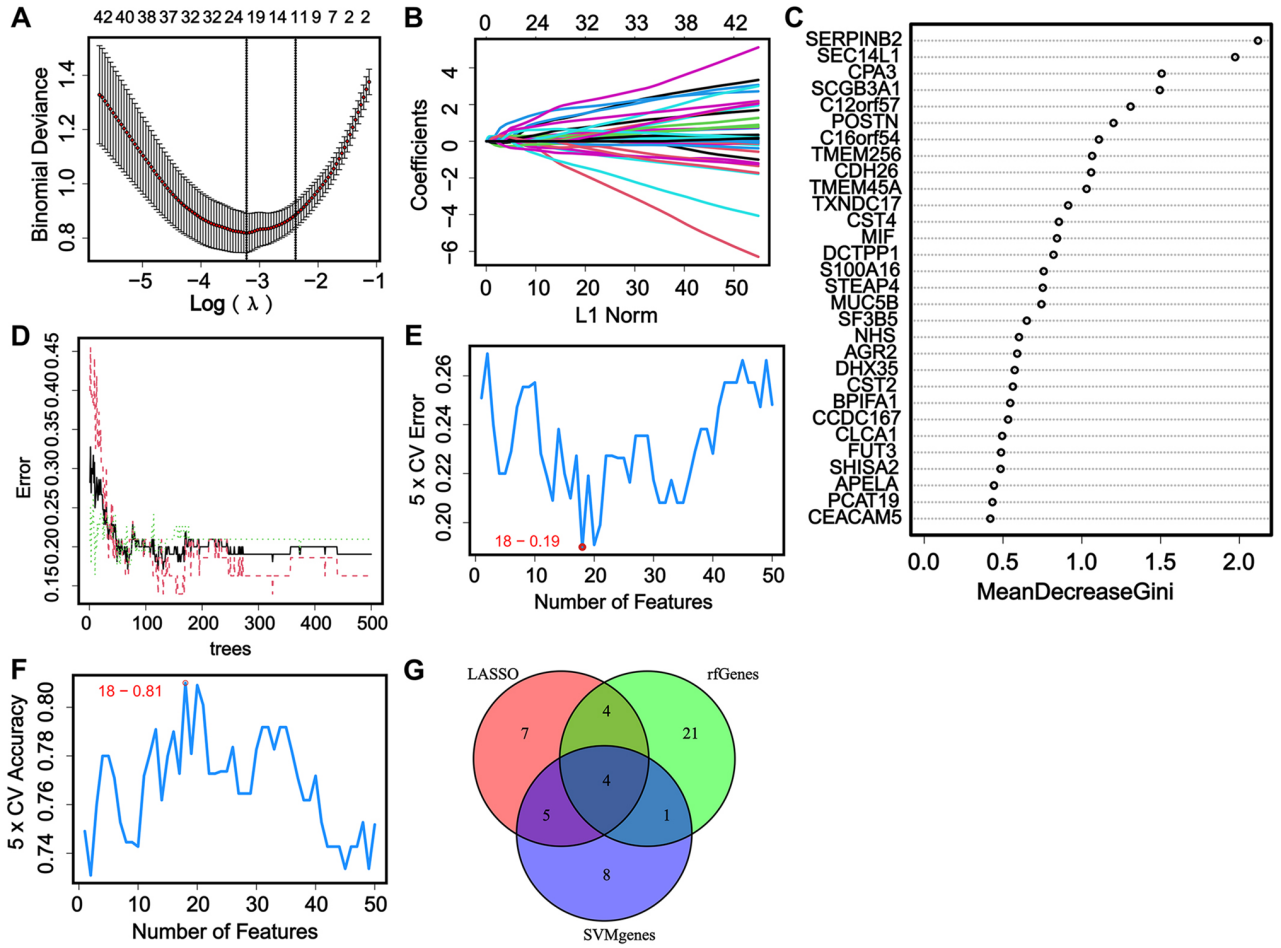
Characterization of hub genes using machine learning algorithms. **A** Least absolute shrinkage and selection operator (LASSO) regression analysis of the module genes; **B** Selection of the optimal penalty parameter for LASSO regression; **C** Random Forest of the module genes, the red represents the asthma samples, the green refers to the healthy volunteers, and the black is for all samples; **D** The most important 30 genes selected with Random Forest; **E**, **F** Support Vector Machine (SVM) characterizing of feature genes; **G** Hub genes were selected by the union of the three machine learning algorithms

The module genes of interest were then trained using LASSO, Random Forest, and SVM algorithms in order to identify the hub genes for asthma. Following normalization to the DEGs, LASSO regression was used to identify the most relevant genes via the minimum criteria (Fig. 3A), and 20 genes were identified as potential hub genes with the optimal lambda value using the LASSO algorithm (Fig. 3B). Random Forest was used to calculate the importance of the module genes of interest. Importantly, the 30 most important genes were chosen as the potential hub genes with mean decrease gini > 0.4 (Fig. 3C). As the number of trees increases, the error rate rapidly decreases. When the number of trees exceeds 30, the decrease in error rate begins to slow down and gradually stabilizes (Fig. 3D). In addition, 18 candidate hub genes were validated using the SVM algorithm, which displayed the lowest 5 × CV error (0.19, Fig. 3E) and highest 5 × CV accuracy (0.81, Fig. 3F). Finally, CCDC167, POSTN, SEC14L1, and SERPINB2 were designated as the asthma hub genes because they were characterized by all three machine learning algorithms (Fig. 3G).

### Validation of the hub genes

**Fig. 4:**
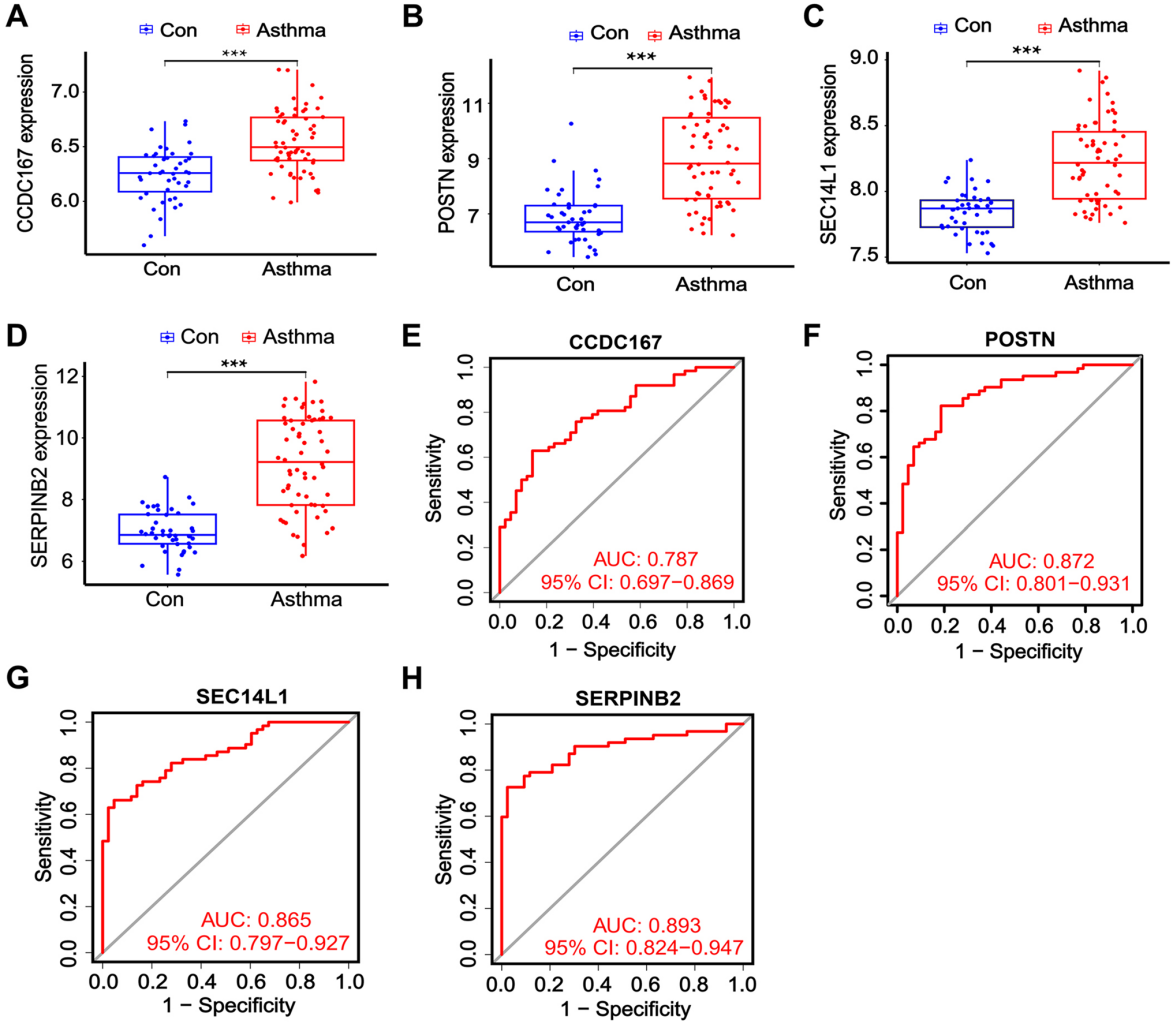
Characterization of the identified hub genes. The levels of the hub genes were standardized to the DEGs, and the expression profiles of the hub genes, including CCDC167 (A), POSTN (B), SEC14L1 (C), and SERPINB2 (D) in the GSE64913, GSE137268 and GSE67472 datasets were evaluated; The predictive value of CCDC167 (E), POSTN (F), SEC14L1 (G), and SERPINB2 (H) was evaluated with ROC curves: ***P < 0.001 by Student’s *t*-test

**Fig. 5:**
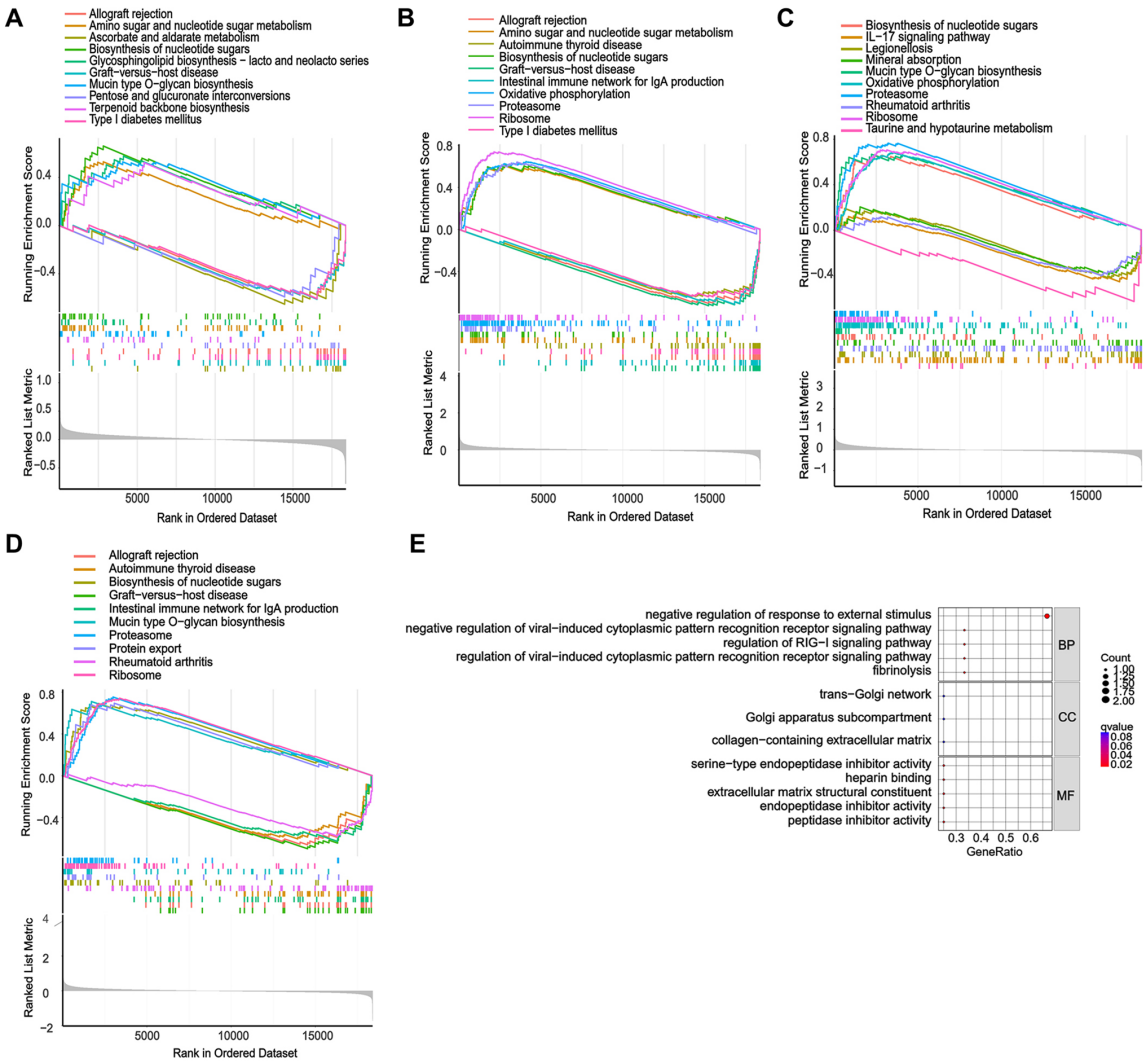
Gene set enrichment analysis (GSEA) and Gene Ontology (GO) enrichment. GSEA of the significant upregulated and downregulated signaling pathways in the hub genes CCDC167 (A), POSTN (B), SEC14L1 (C), and SEPRINB2 (D) in highly expressed individuals. (E) GO enrichment of the hub genes was applied

Next, the hub gene expression levels in the GSE67472 datasets were verified to validate the prediction. The expression of hub genes, including CCDC167, POSTN, SEC14L1, and SERPINB2, was significantly lower in healthy donors (controls) compared to asthma patients (Fig. 4A–D). The predictive value of the hub genes was assessed using ROC curves, and the aero under curves for CCDC167, POSTN, SEC14L1, and SERPINB2 were approximately 0.79, 0.87, 0.86, and 0.89, respectively, supporting their sensitivity and specificity in asthma diagnosis (Fig. 4E–H). The GSEA showed that in patients having a high expression of CCDC167, amino sugar and nucleotide sugar metabolism, biosynthesis of nucleotide sugars, glycosphingolipid biosynthesis—lacto and neolacto series, mucin-type O-glycan biosynthesis, and terpenoid backbone biosynthesis were upregulated. Conversely, allograft rejection, ascorbate and aldarate metabolism, graft-versus-host disease, pentose and glucuronate interconversions, and type I diabetes mellitus were downregulated (Fig. 5A). On the other hand, in patients having a high expression of POSTN, amino sugar and nucleotide sugar metabolism, biosynthesis of nucleotide sugars, oxidative phosphorylation, proteasome and ribosome were upregulated. Conversely, allograft rejection, autoimmune thyroid disease, graft-versus-host disease, intestinal immune network for IgA production, and type I diabetes mellitus were downregulated (Fig. 5B). Moreover, in patients having a high expression of SEC14L1, biosynthesis of nucleotide sugars, mucin-type O-glycan biosynthesis, oxidative phosphorylation, proteasome, and ribosome were upregulated. Conversely, IL-17 signaling pathway, legionellosis, mineral absorption, rheumatoid arthritis, taurine, and hypotaurine metabolism were downregulated (Fig. 5C). Furthermore, in patients with elevated SERPINB2 expression, biosynthesis of nucleotide sugars, mucin-type Oglycan biosynthesis, proteasome, protein export, and ribosome were upregulated. On the other hand, allograft rejection, autoimmune thyroid disease, graft-versus-host disease, intestinal immune network for IgA production, and rheumatoid arthritis were downregulated (Fig. 5D). In addition, the application of GO analysis (Fig. 5E) revealed that the hub genes were enriched in fibrinolysis (BP), regulation of viral-induced cytoplasmic pattern recognition receptor signaling pathway (BP), regulation of RIG-I signaling pathway (BP), negative regulation of viral-induced cytoplasmic pattern recognition receptor signaling pathway (BP), negative regulation of response to external stimulus (BP), collagen-containing extracellular matrix (CC), Golgi apparatus subcompartment (CC), trans-Golgi network (CC), peptidase inhibitor activity (MF), extracellular matrix structural constituent (MF), endopeptidase inhibitor activity (MF), serine-type endopeptidase inhibitor activity (MF), and heparin-binding (MF). These results suggest that the characterized hub genes were upregulated in asthma patients and were involved in disease signaling transduction. Among these enrichments, coiled-coil domain-containing protein 167 (CCDC167) was the gene involved in the greatest number of processes.

### In vivo validation of the hub gene CCDC167

**Fig. 6:**
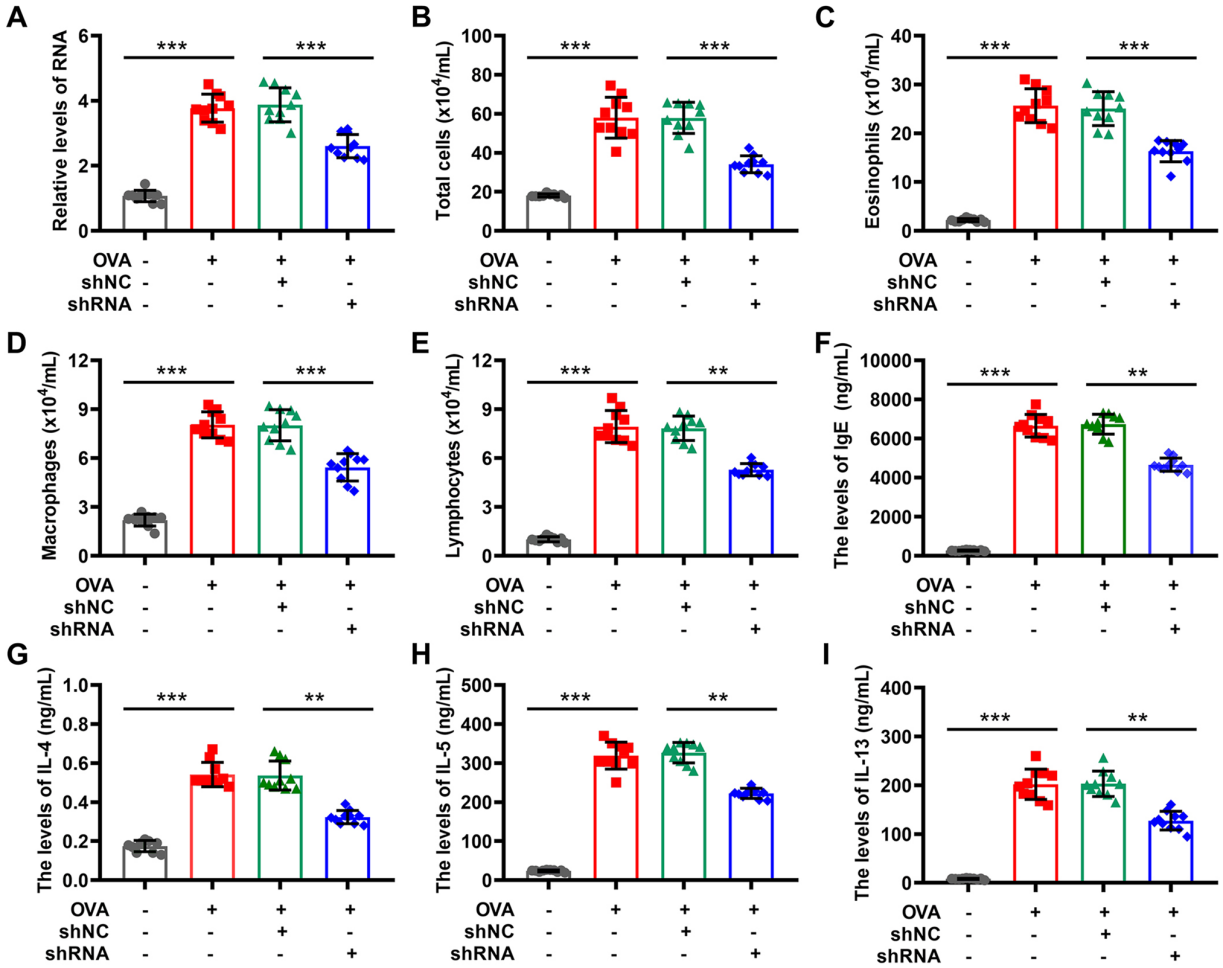
Effect of shCCDC167 on inflammation in asthmatic mice. **A** The silencing of CCDC167 in asthmatic mice was confirmed with qRT-PCR (WB?); **B** the level of total bronchoalveolar lavage fluid (BALF) cells; **C** the level of eosinophils in BALF; **D** the level of macrophages in BALF; **E** the level of lymphocytes in BALF; **F** the level of IgE in BALF; **G** the level of IL-4 in BALF; **H** the level of IL-5 in BALF; **I** the level of IL-13 in BALF. *N* = 10 for each group, ***P < 0.001 by Student’s *t*-test

CCDC167 was upregulated in different types of tumors as previously reported (Chen et al. 2021). However, the role of CCDC167 in the development of asthma still remains unclear. To further study the mechanism of CCDC167 in asthma, he shCCDC167 was used to downregulate the expression of CCDC167 in mice with asthma, and the resultant impacts of shCCDC167 were examined. The suppression of gene expression was confirmed in mice treated with shCCDC167, as demonstrated in Fig. 6A. In comparison to the control group (shNC), the experimental group (shCCDC167) exhibited decreased levels of total cell counts (Fig. 6B), eosinophils (Fig. 6C), macrophages (Fig. 6D), and lymphocytes (Fig. 6E) in BALF. Additionally, the shCCDC167 group demonstrated significantly lower levels of inflammatory cytokines, including IgE (Fig. 6F), IL-4 (Fig. 6G), IL-5 (Fig. 6H), and IL-13 (Fig. 6I), in BALF.

**Fig. 7:**
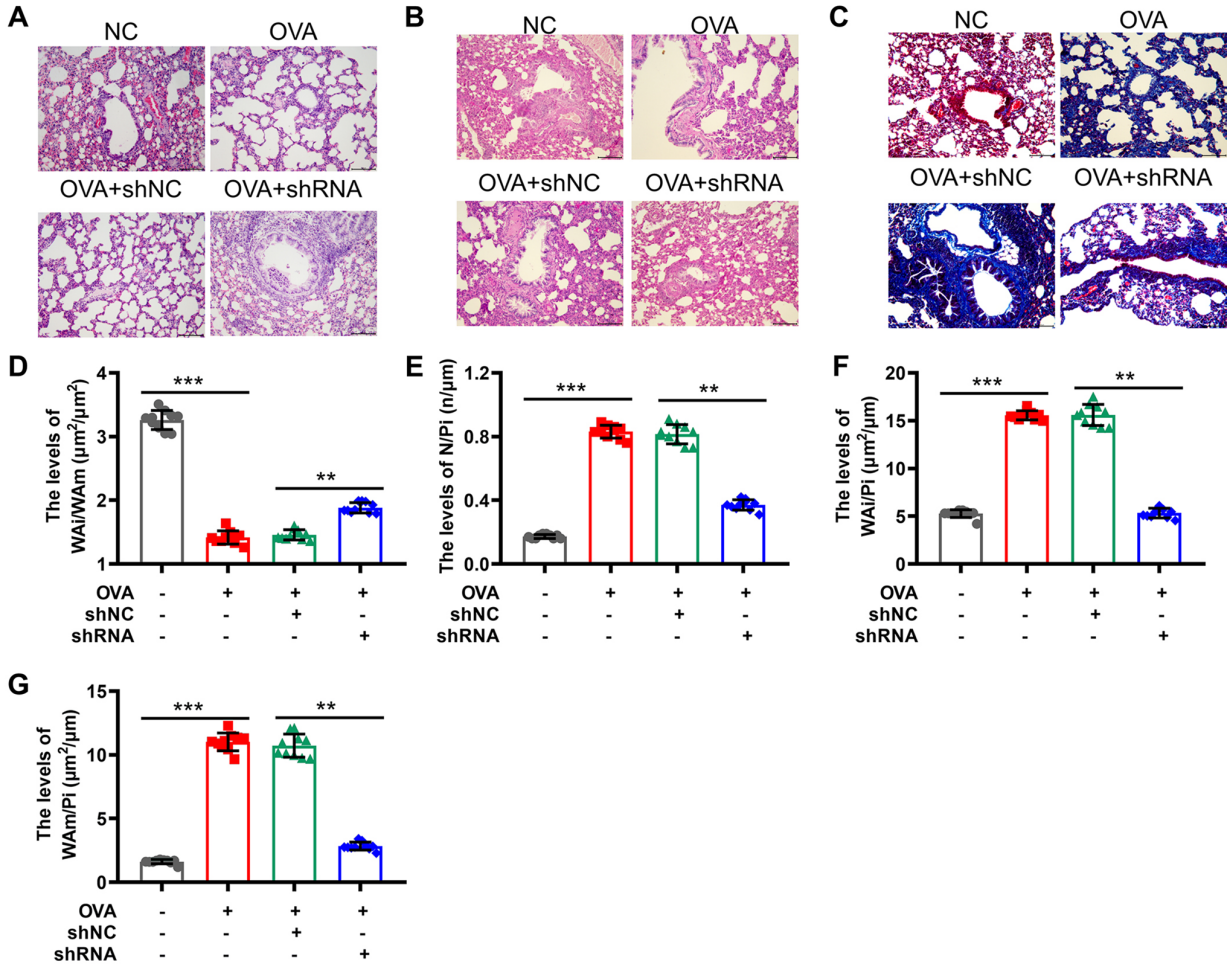
Effect of shCCDC167 on lung tissue damage in asthmatic mice. **A** Inflammatory cell infiltration in asthmatic mice was indicated with H&E staining, scale = 100 μm; **B** pathologic morphology in asthmatic mice tested with Periodic acid-Schiff (PAS) staining, scale = 100 μm; **C** smooth muscle hyperplasia and fibrosis in asthmatic mice visualized with Masson staining, scale = 100 μm; The inner area of the bronchial wall (WAi), airway smooth muscle area (WAm), number of bronchial smooth muscle cells (N), and inner perimeter of the bronchial wall (Pi) were measured with Image Pro Plus, and Wai/WAm (D); N/Pi (E); Wai/Pi (F); and WAm/Pi (G) were calculated. N = 4 for each staining and each of the sections was observed in 4 randomized 400X fields: ***P < 0.001 by Student*'*s *t*-test

Subsequently, the lung histology of the mice was examined. The airways of the shNC mice exhibited significant cellular infiltration and damage to alveolar structures, as observed through H&E staining. However, these effects were mitigated in the shCCDC167 group (Fig. 7A). Goblet cell hyperplasia and the production of airway mucus were detected in shNC mice using PAS staining. However, these effects were notably reduced in the shCCDC167 group, as shown in Fig. 7B. Furthermore, there was a notable presence of smooth muscle hyperplasia and fibrosis in the small airway tissues of the shNC group. However, this observation was reversed in the shCCDC167 group, as depicted in Fig. 7C. In the asthmatic mice treated with shNC, there was a notable reduction in WAi/WAm levels, accompanied by a noteworthy elevation in N/Pi, WAi/Pi, and WAm/Pi levels. However, these changes were reversed upon treatment with shCCDC167, as depicted in Fig. 7D–G. The observations made in vivo provide empirical support for our initial hypothesis that CCDC167 has the potential to serve as both a biomarker and a therapeutic target for asthma.

## Discussion

Asthma has been one of the most prevalent airway diseases in the world, affecting more than 2.3 million people and causing approximately 37,600 deaths in 2016 (2020). Unfortunately, this disease remains incurable, and the treatment has long been “symptom-control” oriented, which requires long-term medication and results in a series of adverse reactions (Moore et al. 2022; Skoner et al. 2022; Skov et al. 2022). Consequently, the identification of novel biomarkers and therapeutic targets for the treatment of asthma is urgent and essential. With the inclusion of datasets of epithelial samples and induced sputum samples, as well as the application of different training algorithms, we aimed to reduce the influence of heterogeneity in the current study. Four hub genes, CCDC167, POSTN, SEC14L1, and SERPINB2, have been characterized and confirmed with training databases and in vivo.

Asthma is obviously a heterogeneous disease with its many phenotypes, such as allergic and non-allergic, T2 subtype, eosinophilic, and endotypes defined with different immunological mechanisms (Calvén et al. 2020). Therefore, it is difficult to characterize asthma biomarkers and therapeutic targets. In addition, asthma is stimuli-sensitive, which could potentially lead to heterogeneity in clinical samples under varying environmental conditions, such as humidity and air pollution of the air (Lam et al. 2016; Strauss et al. 1978). Inclusion of specific subtypes and restriction of the environment may increase consistency and improve the quality of analyzed hub genes but may limit their applicability to other subtypes of asthma. In addition, over-subdivided phenotypes would cause difficulties in drug development and clinical decision-making. Several types of samples were collected for asthma in different researches, including exhaled air, airway epithelial biopsies, induced sputum, serum, and urine (Popović-Grle et al. 2021). Among these samples, airway epithelial biopsies and induced sputum were generated directly from the infection focus, and are therefore expected to more accurately represent the pathology of asthma patients, such as cough, wheezing, shortness of breath,etc. (Aegerter and Lambrecht 2023; Bakakos et al. 2011; Bradley et al. 1991). Asthma refers to abnormalities of immune cells, the epithelium of the airways, and their interactions (Calvén et al. 2020). Both airway epithelial biopsy and induced sputum were important diagnostic samples involving airway epithelial cells and inflammatory cells at different levels (Maestrelli et al. 1995). In the present study, we demonstrated that DEGs for epithelial biopsy and induced sputum were sufficiently distinct to ensure relevance and heterogeneity and included DEGs from both the epithelium and immune cells.

The utilization of Weighted WGCNA and machine learning algorithms has been employed in the characterization of hub genes associated with various diseases, including asthma. WGCNA has the ability to identify and characterize gene modules that are most pertinent to specific traits (Li et al. 2021). This allows for the exclusion of DEGs that may be statistically significant but not relevant to the traits under investigation. In their study, Yan Li et al. conducted an analysis of DEGs in the GSE64913 dataset, and characterized the asthma-related traits within the same dataset. Their findings demonstrated that ANXA8, ATF4, CD44, CYCS, DDIT3, FKBP5, LDHA, PMAIP1, S100A2, and SFN exhibited potential as hub genes associated with asthma (Li et al. 2023). Although the majority of their predicted genes demonstrated limited prognostic value when referencing the same dataset, this finding underscores the limited efficacy of hub gene characterization solely through the use of WGCNA. In their study, Ding et al. employed the WGCNA technique along with five machine learning algorithms to identify hub genes associated with lipid metabolism. Their findings indicated that CH25H exhibited potential as a biomarker for asthma in relation to lipid metabolism (Ding et al. 2023). Accordingly, the inadequacy of a singular biomarker for this diverse disease was apparent. In this study, we have identified four hub genes that are associated with asthma. Previous research has demonstrated that two of these genes, namely POSTN and SERPINB2, are considered T2 signature genes and have shown promise as potential biomarkers (Burgess et al. 2021; Du et al. 2022; Mo et al. 2019). However, the functional roles of CCDC167 and SEC14L1 in the context of asthma have yet to be fully elucidated.

POSTN is a well-characterized biomarker and crucial asthma regulator. POSTN promoted mucin hypersecretion and sustained eosinophilic inflammation, both of which were essential asthma pathologies of asthma (Burgess et al. 2021). POSTN was also a biomarker and a key player in tissue remodeling and fibrosis, which are important processes in bronchial asthma (Izuhara et al. 2015). SERPINB2 has been identified as an adaptive immunity regulator. Compared to wild-type SERPINB2(+/+) macrophages and mice, OVA significantly induced OVA-specific IFN-gamma-secreting T cells and enhanced the secretion of Th1-promoting cytokines in SERPINB2(−/−) macrophages and mice (Schroder et al. 2010). SERPINB2 was positively correlated with the severity of bronchial asthma (NE et al. 2017), and antagonizing SERPINB2 could inhibit the differentiation of Th2 cells in vitro (Zhou et al. 2021). Our analysis strategy identified POSTN and SERPINB2 as hub genes, which highlighted the significance of the two genes and supported the efficacy of our analyses.

As determined by GSEA, the hub genes were primarily enriched with the biosynthesis of nucleotide sugars, mucin-type O-glycan biosynthesis, proteasome, and ribosome, whereas they were negatively correlated with allograft rejection and graft-versus-host disease. CCDC167 was associated with the majority of these predicted biological processes, emphasizing its central role in asthma. However, CDC167 was previously an uncharacterized asthma gene. CCDC167 was reported to be significantly upregulated as a hub gene in the lungs of chloroprene-treated mice (Guo and Xing 2016), and to be downregulated by multiple antitumor therapeutics in breast cancer patients (Chen et al. 2021). In the present study, CCDC167 was silenced in OVA-induced asthmatic mice, and the inhibitory effect of CCDC167 silencing in asthma was confirmed by decreased inflammatory cytokines and an improvement in airway injuries.

## Conclusion

The four hub genes, namely CCDC167, POSTN, SEC14L1, and SERPINB2, were subjected to characterization and validation using the training databases. In particular, the role of CCDC167 as a crucial factor and a possible therapeutic target for asthma was confirmed through experimentation on mice with OVA-induced asthma.

## Data Availability

The datasets obtained and analyzed during the current study were made available from the corresponding authors through request. Gene expression data (GSE67472, GSE64913 and GSE137268) were downloaded from the Gene Expression Omnibus (http://www.ncbi.nlm.nih.gov/geo).
